# Localization Matters: Epigenetic Regulation of Natural Killer Cells in Different Tissue Microenvironments

**DOI:** 10.3389/fimmu.2022.913054

**Published:** 2022-05-30

**Authors:** Gabriela M. Wiedemann

**Affiliations:** Department of Internal Medicine II, Klinikum rechts der Isar, Technical University of Munich, School of Medicine, Munich, Germany

**Keywords:** NK cell, epigenetic regulation, histone modification, tumor microenvironment, tissue microenvironment, cancer immunology

## Abstract

Natural Killer cells (NK cells) are cytotoxic innate lymphoid cells (ILCs), which play a key role in the early protection against viral infection and cancer. In addition to mounting rapid effector responses, NK cells possess the capacity to generate long-lived memory cells in response to certain stimuli, thus blurring the lines between innate and adaptive immunity and making NK cells an ideal candidate for tumor immunotherapy. NK cell development, activation and memory formation are regulated by epigenetic alterations driven by a complex interplay of external and internal signals. These epigenetic modifications can convey long-lasting functional and phenotypic changes and critically modify their response to stimulation. Here, we review how NK cell functionality and plasticity are regulated at the epigenetic level in different tissue microenvironments and within tumor microenvironments. An in-depth understanding of the epigenetic modifications underlying NK cell functional diversity in different environments is an essential step in the development of NK cell-based cancer therapies.

## Introduction

Natural Killer cells (NK cells) are innate lymphoid cells (ILCs) characterized by the capacity to rapidly recognize and lyse target cells without prior sensitization. Moreover, NK cells possess the ability to mount a long-lived antigen-specific memory response upon certain stimuli (1). These features make NK cells critical effector cells in the antiviral and antitumoral immune response, and have led to great interest in strategies to harness NK cells for tumor immunotherapy (2–4). Despite its swiftness, NK cell activation and target cell recognition is a complex multistep process. Since NK cells are not capable of somatic DNA rearrangement, they rely on a repertoire of germline-encoded inhibitory and activating receptors for target cell recognition (5). Activating NK cell receptors typically recognize stress-induced or virally encoded ligands on malignant or infected cells (6). Inhibitory NK cell receptors recognize MHC-I molecules, thereby preventing the killing of healthy cells, but also triggering activation through lack of inhibition when MHC-I is downregulated or missing on aberrant cells (7). Besides receptor ligation, NK cells require additional signals for maximal activation, namely proinflammatory cytokine signals and co-stimulation. Convergence of these diverse signals leads to transcriptional and epigenetic changes within the NK cell, leading to short-term and/or long-term changes in functionality (8). The consequences of these signals are influenced by the NK cell’s maturation and differentiation status as well as by the interaction with external signals present in the local microenvironment. Thus, NK cells residing in tissues can display diverging functional characteristics from conventional blood-circulating NK cells and especially tumor-infiltrating NK cells often display severely impaired functionality (9–11). Changes in the local microenvironment, for example within and around solid tumors, may therefore critically affect NK cell functions on many levels. Since epigenetic changes can be persistent, thereby leading to stable fate decisions, it is important to understand how the diverse signals, which NK cells encounter locally and systemically, interact on a transcriptional and epigenetic level. Unravelling these questions will help us to better understand the determinants of NK cell functionality in different tissues and provide a basis for the development of NK cell-based therapies for solid cancers.

We recently reviewed how NK cell activation and memory formation are epigenetically regulated (12). The aim of this review is to provide an overview of how NK cell phenotypic and functional plasticity is epigenetically regulated during development and in different microenvironmental contexts. First, we will address how lineage commitment, maturation and key functions are controlled by epigenetic mechanisms. Second, we will recapitulate our current understanding of how different tissue microenvironments, as well as the tumor microenvironment, shape the epigenetic landscape and thereby phenotypic and functional properties of NK cells and ILCs.

## Modes of Epigenetic Regulation

**Figure 1:**
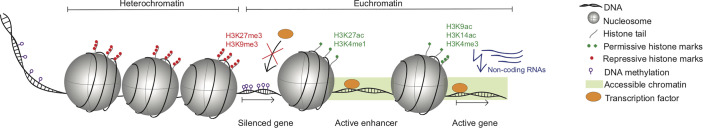
Schematic of different modes of epigenetic regulation. Chromatin in the nucleus is found in two major states: the tightly condensed heterochromatin, and the more lightly packed euchromatin, which is more accessible for transcription. Both in euchromatin and heterochromatin DNA is wrapped around nucleosomes, which are octameric complexes consisting of eight histone proteins. Within this chromatin architecture, different factors regulate the accessibility of the DNA for transcription factors: histone proteins can undergo post-translational modifications, which influence chromatin structure and accessibility in different ways. Histone marks H3K27me3 and H3K9me3 are associated with transcriptional repression, whereas the permissive histone marks H3K27ac and H3K4me1 are found on enhancers and H3K9ac, H3K14ac and H3K4me3 are found on active promoters. On the DNA level, methyl residues dynamically influence the transcriptional activity, whereby methylation of promoters is generally considered to repress transcription. Additionally, non-coding RNAs like miRNAs and lncRNAs can influence gene transcription on diverse levels, thereby adding another layer of epigenetic regulation.

Epigenetics refers to the diverse mechanisms controlling gene transcription, which are independent of the DNA sequence itself and are therefore “above” (*epi)* the genetics. These mechanisms are key to enabling diversity in genetically identical cells, thereby proving critical to the development and differentiation of different tissues in the body. In NK cells, epigenetic changes enable a remarkable heterogeneity in expression of genome-coded receptors across an individual’s NK cell populations (13). One of the first descriptions of epigenetic regulation was introduced in the 1970s with the discovery of DNA methylation (14). Since then, our understanding of the epigenetic machinery has broadened and an increasing number of modes of epigenetic regulation have been defined, including DNA methylation, histone modifications, chromatin accessibility and non-coding RNAs. We will give brief overview of some of these mechanisms here (**Figure 1**).

### DNA Methylation

DNA methylation denominates the transfer of a methyl residue to a cytosine or adenine base in the DNA (15, 16). This process is highly conserved and known to play a role in X chromosome inactivation, genomic imprinting, embryonic development and in cancerogenesis, amongst others (16–21). DNA methylation is executed by DNA methyl transferases (DNMT), and most of it takes place on CpG dinucleotides, with 70 – 80% of CpG sites in the human genome being methylated, whereas demethylation is carried out by Tet enzymes (22–27). Generally, DNA methylation at promoters is considered to repress transcription (28, 29). Moreover, DNA methylation and DNMT action have been described to interact with other modes of epigenetic modification, like the deposition of histone marks and the enzymes conveying these marks (30). In immune cells, DNA hypomethylation of functional target genes has been described upon activation or functional differentiation (31), like the hypomethylation of the *IFNG* locus in human adaptive NK cells (32). Furthermore, a genome-wide study of DNA methylation in NK cells, ILC2 and ILC3 not only showed a clear segregation between NK cells and ILC with regards to DNA methylation, but also a high correlation between methylation status, gene expression and the abundance of certain histone modifications (33).

### Histone Modifications

Chromatin in the nucleus is organized in nucleosomes, which are small structural units in which the DNA is wrapped around an octameric complex consisting of two of each histone molecules H2A, H2B, H3 and H4. Posttranslational modifications to these histones have first been described in 1964 by Allfrey et al. and since then new modifications and novel functions of these modifications have steadily been discovered (34). Histone modifications can be of diverse nature, with the most prevalent modifications being methylation and acetylation. Other modifications include phosphorylation, ubiquitinylation, sumoylation, ADP ribosylation, deamination, propionylation and butyrylation (35, 36). Upon their first discovery, histone modifications were thought to play a role in the interaction of the DNA with the nucleosomes by changing the charge of the histones. We now know that the functions of histone modifications are diverse, including changes in chromatin compactness and accessibility and the recruitment of transcription factors, transcriptional activators and other effector proteins (37–39). In immune cells, histone modifications have been shown to play a role in the cellular differentiation at steady state as well as during the immune response (40, 41). We now want to give a short introduction into the two most prevalent and well-studied histone modifications: methylation and acetylation.

#### Histone Methylation

Out of all four histones, H3 is the primary site of methylation processes and methylation takes place predominantly on lysine (K) or arginine (R) residues (42). There are three different histone methylation states, which are controlled by the activities of histone methyl transferases and histone demethylases: mono-methylation (m1), di-methylation (m2) or tri-methylation (m3). Whether histone methylation confers a permissive or repressive function is context-dependent. For example, histone tri-methylation at K4 of histone H3 (H3K4me3) is a permissive mark often enriched on active promoters, whereas mono-methylation of the same K residue (H3K4me1) is an activating mark found on enhancers (43, 44). In contrast, tri-methylation of K9 and K27 on H3 (H3K9me3 and H3K27me3) are repressive histone marks, with H3K9me3 marking heterochromatin and H3K27me3 being enriched around silenced genes, thus playing a critical role in the repression of developmental genes (45, 46). Interestingly, the repressive mark H3K27me3 and the permissive mark H3K4me3 have been described to co-occur on so-called bivalent promoters, where they mark developmentally regulated genes and account for a chromatin state which is currently transcriptionally inactive but poised for activation (47, 48). In NK cells, changes in H3K4me3 deposition can be observed in the course of MCMV infection and are highly correlated to the H3K4me3 landscapes upon cytokine stimulation (49).

#### Histone Acetylation

Like methylation, histone acetylation is mostly found on K residues of H3, and it’s carried out by histone acetyltransferases (HATs), which transfer acetyl groups from acetyl CoA to the histone tails. Nuclear HATs are divided into the GNAT, MYST and p300/CBP families (50). Conversely, histone deacetylases (HDACs), which are subdivided into four groups (HDAC I-IV), can remove acetyl groups. Since acetylated histones are generally associated with a chromatin state permissive to transcription, HDAC activity can therefore negatively impact transcriptional activity. Acetylation of K27 on H3 (H3K27ac) is enriched on active enhancers, whereas H3K9ac and H3K14ac accumulation is found on active promoters but has also been described to enrich in active enhancer regions (51–53). Moreover, the exclusive occurrence of H3K14ac on promoters has been described to mark crucial tissue-specific genes in *Drosophila (* 53 *).* In NK cells, H3K27ac deposition on enhancer regions is dynamically modified by inflammatory cytokines and their downstream transcription factors, thereby regulating gene expression during the immune response (12). Intriguingly, acute activation leads to a rapid (< 6 h) and massive re-shaping of the NK cell enhancer landscape, enabling the transcription of highly induced genes and demonstrating the importance for epigenetic remodeling in the early immune response to infection (54). In the context of cytokine stimulation, the establishment of *de-novo* enhancers is dependent on the interleukin-12 downstream transcription factor (TF) STAT4, clearly demonstrating how TFs interact with the epigenetic landscape to regulate transcription (54).

### Chromatin Accessibility

Only 2-3% of the genomic DNA is accessible. However, the vast majority (94.4%) of TF binding measured by the ENCODE project occurs within these few accessible regions (55). Strategies to measure chromatin accessibility generally rely on the susceptibility of the DNA to cleavage enzymes (56). The currently most widely used technique is assay for transposase-accessible chromatin sequencing (ATAC-seq), which uses a hyperactive Tn5 transposase to simultaneously cleave and tag accessible regions (57). Chromatin accessibility is mainly influenced by nucleosome density and turnover as well as by the allocation of other chromatin-binding proteins and it has been shown to be dynamically regulated during development and differentiation and in response to external stimuli (56, 58, 59). In NK cells as well as in T cells, transient and stable changes in chromatin accessibility have been demonstrated to guide their transition from naïve to effector and memory cells (59).

### Non-Coding RNAs

About 98% of RNAs transcribed in humans are not protein-coding (60). These non-coding RNAs comprise different molecules, including microRNAs (miRNAs), transfer RNAs (tRNAs), ribosomal RNAs (rRNAs), long non-coding RNAs (lncRNAs) and circular RNAs (circRNAs) (61). Of these, miRNAs and lncRNAs are best described in their functions as epigenetic regulators. MiRNAs result from the cleaving of pre-miRNAs, resulting in an approximately 22 bp long mature miRNA duplex (62, 63). These mature miRNAs bind to target mRNA with the help of the RNA-induced silencing complex (RISC), where they suppress its translation or provoke its degradation in a sequence-specific manner (64). LncRNAs, by contrast, are defined by their size of more than 200 nucleotides (65). The diverse mechanisms with which lncRNAs execute their gene regulatory functions are still under investigation. So far, it is known that lncRNAs can regulate histone modifications like acetylation and methylation, impact DNA methylation, directly interfere with gene transcription by binding to DNA sequences or interaction with TF as well as participate in post-transcriptional regulation like splicing and interaction with miRNAs (66). During the NK cell memory response to murine cytomegalovirus (MCMV), the miRNA miR-21 was shown to regulate the proliferative burst in the early days of infection (67).

## Epigenetic Regulation of NK Cells

**Figure 2:**
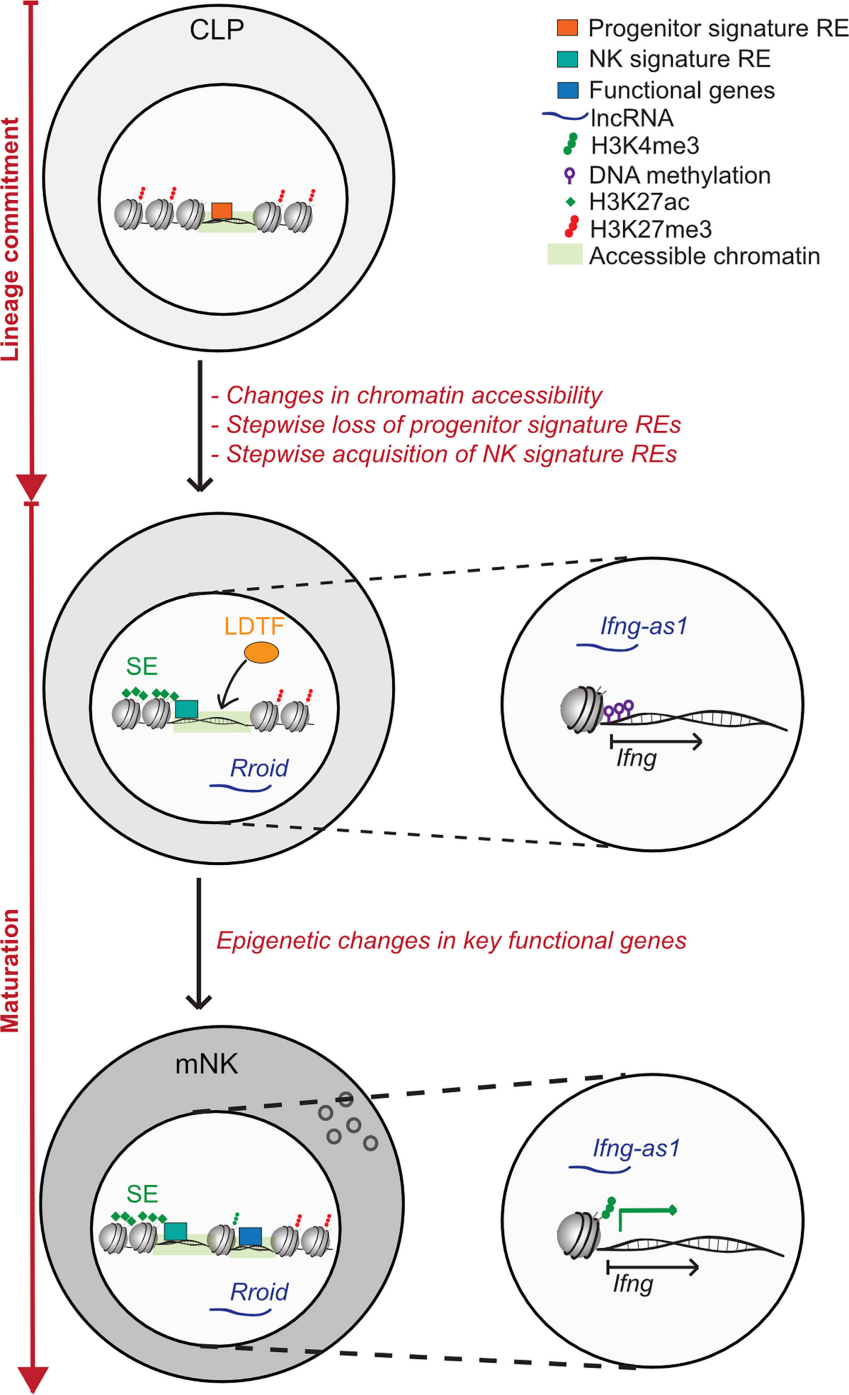
Epigenetic regulation of NK cell lineage commitment and maturation. Lineage commitment: NK cells develop from a common lymphoid progenitor (CLP) in a multistep process, of which several steps are excluded in this schematic. During lineage commitment, NK cells were found to undergo striking changes in chromatin accessibility. Regulatory elements (RE) defined by epigenetic features show a highly dynamic re-organization during lineage-commitment: the stepwise loss of progenitor signature REs on genes like PU.1 is accompanied by the acquisition of NK cell signature REs. Of these, cell-type specific REs are often marked by super enhancers (SEs), which are found on genes crucial to cell identity and functionality. Areas, which display an increase in chromatin accessibility during lineage commitment are often enriched in lineage-defining transcription factor (LDTF) binding sites. Moreover, the lncRNA Rroid, which regulates the NK cell LDTF ID2 was shown to promote both lineage commitment and maturation in NK cells and ILC1. NK cell maturation: During NK cell maturation, further functional specialisation is promoted on an epigenetic level. In human NK cells, terminal maturation is accompanied by an extensive epigenetic remodelling of the *Ifng* locus, which consisted of consecutive demethylation of the *Ifng* transcriptional start site (TSS) and the acquisition of the permissive histone mark H3K4me3, both leading to a more effective IFN- γ production. IFN- γ transcription in mature NK cells is further regulated by the lncRNA *Ifng-as1*, which regulates local and distal chromatin interactions with the *Ifng* locus.

**Figure 3:**
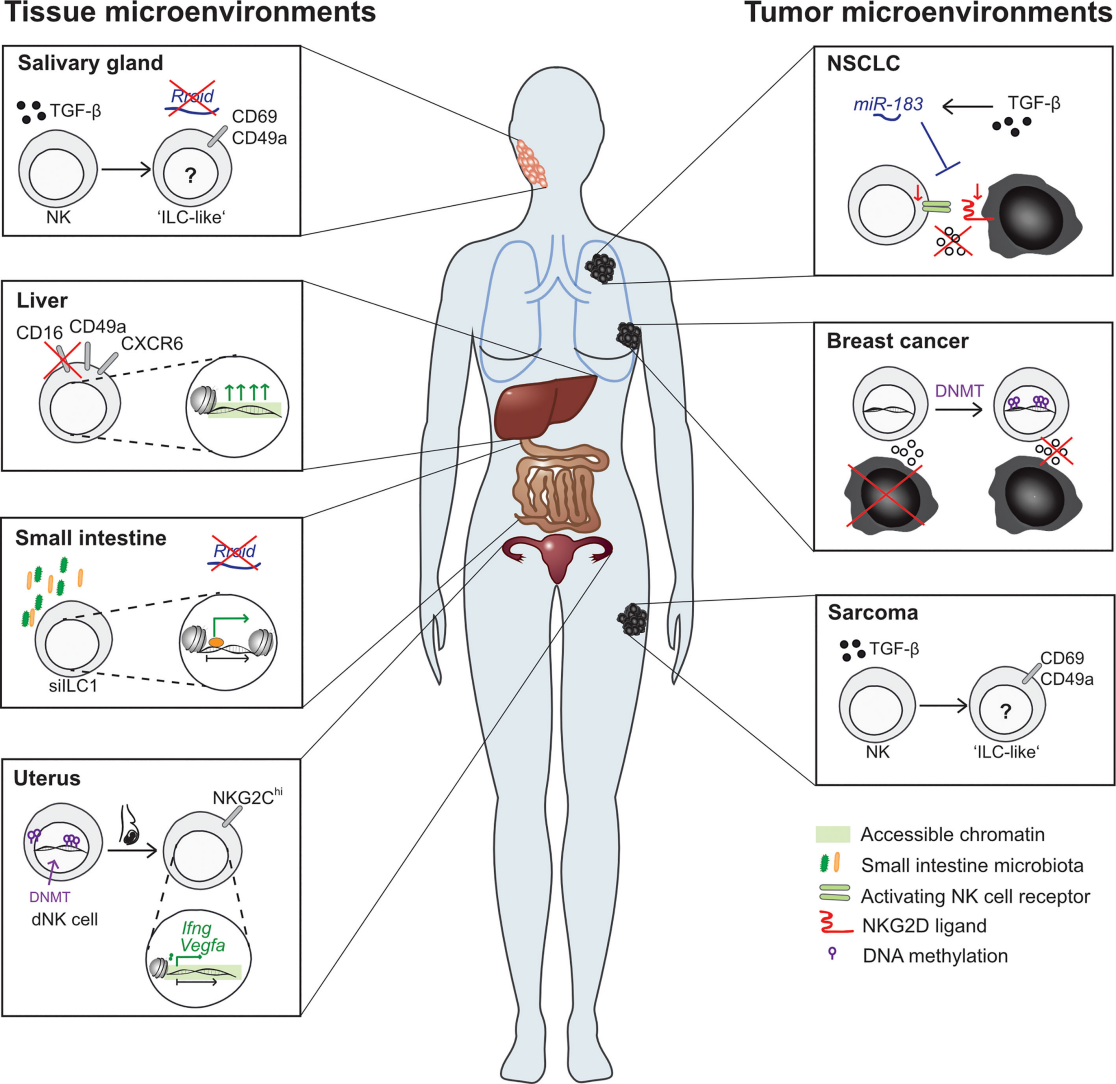
Effects of different tissue microenvironments on NK cell and ILC epigenetic features. Tissue microenvironments (left side): NK cells in different tissues display strikingly unique features. For example, NK cell numbers in the salivary gland and small intestine are independent of the presence of the lnc-RNA *Rroid*, which is required for NK cell and ILC1 development in other tissues. Moreover, the enrichment of TGF-β in the salivary gland microenvironment leads to the conversion of NK cells into an ILC1-like phenotype. In the liver, tissue-resident NK cells display a unique phenotype: CXCR6-expressing NK cells are most abundantly found in the liver as compared to other tissues and display singular functional features. In human livers, a CD49a^+^ CD16^-^NK cell subsets is marked by disparate chromatin accessibility patterns which regulate antigen-specific cytotoxicity. Within the small intestine, NK cells and ILC display unique phenotypic and transcriptional features, separating them from NK cells from other tissues. The role of local microbiota in shaping this phenotype is underlined by the fact that antibiotic treatment leads to marked alterations in the epigenetic landscape of small intestine ILC1. In the uterus, a functionally and phenotypically unique subset of NK cells with reduced cytotoxicity is found. Decidual NK cells (dNK cells) show divergent DNA methylation patterns and pregnancy leads to the development of a NKG2C^hi^ NK cells subset with increased chromatin accessibility at the *Ifng* and *Vegfa* locus. Tumor microenvironments (right side): Malignant tissues also provide severely altered microenvironments, which affect NK cells on multiple levels. NSCLC-infiltrating NK cells display decreased DAP12-expression, caused by TGF-β induced miR-183. MiR-183 targets both DAP12 in NK cells, leading to a decrease in activating receptor signaling and cytotoxicity, and NKG2D ligands in cancer cells, leading to a further decrease in NK cell recognition. Contact of NK cells with breast cancer cells leads to a functional switch in NK cells from tumoricidal to metastasis-promoting, which was shown to be associated with DNMT upregulation. In MCA sarcomas, high levels of TGF-β leads to a conversion of NK cells into ILC1-like cells with lower anti-tumoral potential.

NK cell activation and memory formation are dependent on proinflammatory cytokine signals. These cytokine signals lead to vast transcriptional and epigenetic changes and they interact on multiple levels (49, 68–71). Monitoring of chromatin accessibility throughout the course of NK cell memory formation unveiled dynamic changes in the epigenetic landscape, some of which are only transient during the effector response, but some of which are long-lasting, leading to a sustained functional commitment of the NK cell (59). The exact epigenetic mechanisms governing NK cell activation and memory formation were recently reviewed elsewhere (12). However, there are further aspects in NK cell biology, where transient and persistent epigenetic changes can relevantly change the cell’s fate regarding both differentiation and functionality. One of these aspects is NK cell development, where critical steps at developmental branches are epigenetically controlled. In addition, NK cell phenotype and function are also strongly dependent on their localization, and signals conveyed by the tissue microenvironment are likely to have an effect on the epigenetic programs in NK cells as well. Here, we aim to gather what is known on how epigenetic mechanisms regulate NK cell lineage commitment decisions and crucial functional programs (**Figure 2**). Secondly, we want to shed light on how different tissue and tumor microenvironments influence the epigenetic landscape in NK cells (**Figure 3**).

### Epigenetic Regulation of NK Cell Differentiation and Functionality at Steady State

NK cell maturation and differentiation is a multistep process, in which NK cells develop from a common lymphoid progenitor (CLP), which is shared with other ILCs, B and T lymphocytes (72). Shortly after the CLP stage, NK cells branch off into a developmentally separate road, and their further differentiation is controlled by lineage-defining transcription factors (LDTF), including Tox, Nfil3, Id2, Ets1, T-bet and Eomes (72–78). Importantly, the steps leading to lineage commitment are governed by dynamic changes in the epigenetic landscape of NK cells. Investigating chromatin marks in differentiating cells during hematopoiesis, Lara-Astiaso et al. found that lineage commitment was accompanied by the gain and loss of lineage-specific regulatory elements (REs), with a striking 90% of enhancers changing their accessibility and activation state during hematopoiesis (58). Interestingly, lineage-specific enhancers were found to be established in early progenitors, revealing the cell’s future commitment long before the transcriptional program was started (58). Of the enhancers that gained activity during differentiation, a high number displayed motifs for LDTF (58). Accordingly, Shih et al. revealed a stepwise loss of hematopoietic stem cell (HSC)- signature REs, accompanied by the acquisition of NK cell signature REs during NK cell maturation (79). Herein, REs lost during differentiation were enriched for motifs of early progenitor TFs like PU.1, whereas newly acquired REs were enriched for LDTF motifs (79). Along these lines, T-BET and EOMES-bound genes were shown to be significantly more accessible in mature NK cells than in NK precursors, indicating again the concerted action of chromatin conformation and LDTF activity during NK cell development and differentiation (80). Moreover, during the *in vitro* differentiation of NK cells from umbilical cord blood, dynamic changes in chromatin accessibility were observed, with genes encoding LDTF gaining accessibility throughout the course of differentiation, demonstrating that not only binding sites but also the transcription of LDTF is epigenetically regulated (81). Apart from histone modifications and chromatin accessibility, Mowel et al. described the lncRNA *Rroid*, which regulates expression of the LDTF Id2 and promotes lineage commitment, maturation and functionality specifically in group 1 ILCs (containing NK cells and ILC1) but not in ILC2 or ILC3 (82). Altogether, these data make it clear that NK cell lineage commitment and differentiation are controlled by epigenetic mechanisms on multiple levels.

Since epigenetic modifications control cell identity and functionality, the epigenetic landscape of a cell can also be used to deduce cellular attributes. In this context, one particular subtype of REs, which can serve to identify central cell type-specific genes, should be mentioned: super enhancers (SEs). SEs are characterized by a broad deposition of H3K27ac and an enrichment for LDTF binding sites and they are known to regulate the expression of crucial cell identity genes (83, 84). A study investigating the regulomes of T helper (Th) cells versus (vs.) ILCs found > 30% of SEs and only about 5% of conventional enhancers to be cell-type specific (84). Cell-type specific SEs often reflected key functional properties, like the *Prf1*-associated SE in cNK cells, which was absent in ILC1 and reflects the cytolytic competence of NK cells vs. ILC1. However, in recent years the dogma of non-cytotoxic ILC1s has been challenged by the findings of highly cytotoxic, granzyme expressing ILC1-like cells and ILC1 (84–88). Similarly, in a different study, investigation of hyper-accessible enhancers in CD56^bright^ and CD56^dim^ blood NK cells vs. ie ILC1 and ILC3 depicted a clear separation of cells based on 1) their identity (ILC vs. NK cell) and 2) their cytotoxic potential (ILC3 < CD56^bright^ < ieILC1 < CD56^dim^) (89). Again, SE analysis showed a high proportion of SEs to be cell type-specific, and the search for SEs identified novel functionally relevant genes in specific cell types like the G protein-coupled receptor (GPR) EBI2 in CD56^bright^ NK cells, which was shown to play a role in oxysterole-mediated functional inhibition of these cells (89). These data highlight how both cell identity and key functions can be inferred from epigenetic characteristics.

One hallmark of NK cell functionality, which is known to be regulated by multiple epigenetic mechanisms, is interferon gamma (IFN- γ) production. Luetke-Eversloh et al. observed in 2014, that mature CD56^dim^ NK cells more efficiently produce IFN- γ upon activating receptor stimulation than their CD56^bright^ counterparts, a finding that seemingly contradicts the notion that terminally differentiated NK cells produce less cytokine and are more cytotoxic (90). This increase in IFN- γ efficiency was not due to an upregulation of activating receptors, but was caused by epigenetic remodeling of the *Ifng* promoter: during terminal differentiation, the *Ifng* transcriptional start site was shown to be successively demethylated and at the same time acquired the permissive histone mark H3K4me3, thereby enabling transcriptional activation (90). Another critical regulator of IFN- γ production is the lnc-RNA locus *Ifng-as1*, which is found in proximity to the *Ifng* locus itself and the loss of which was demonstrated to significantly reduce IFN- γ expression in T and NK cells (91). The mechanisms by which *Ifng-as1* acts to regulate IFN- γ expression are diverse and include a role for *Ifng-as1* as a chromatin organizer and the underlying locus as a cis-regulatory element (91).

Moreover, a broader functional impact of histone modifications on NK cells has been highlighted in studies examining histone methyl transferases and demethylases: the H3K27 methyl transferase Ezh2 modulates NK cell activating receptor expression and cytotoxicity, whereas H3K27 demethylases regulates pro-inflammatory cytokine production in NK cells (92, 93). Additionally, the H3K4me3 demethylase Kdm5a regulates NK cell activation *via* H3K4me3 demethylation at the promoter region of the inhibitory *Socs1* gene (94). We are only beginning to understand the multiple levels on which different epigenetic signals interact to shape NK cell responses. A more profound investigation of these regulatory networks will be a crucial step in the development of NK cell-mediated therapies.

### Tissue-Specific Effects on the Epigenetic Landscape in NK Cells

NK cells are a heterogeneous cell population, with striking phenotypic and functional diversity depending on their maturation and differentiation status as well as their localization (e.g. tissue-resident NK cells vs. circulating NK cells). Together with ILC1, NK cells are members of group 1 ILCs. The phenotypic differentiation between NK cells and ILCs within tissues is often ambiguous and rendered even more difficult by plasticity in between ILC subgroups and tissue-specific cell heterogeneity (95, 96). However, in recent years, single cell RNA-sequencing studies of human and mouse NK cells and ILCs have contributed tremendously towards a deeper understanding of transcriptional, functional and phenotypic determinants of NK cell and ILC identity in different tissues, again highlighting the diversity of these populations (87, 97–99). Since NK cell maturation, differentiation and functionality are essentially regulated by epigenetic mechanisms, as discussed above, the question arises, what are the tissue-specific cues that impact the epigenetic landscape and thus the functional properties of tissue-resident NK cells and other ILCs. Despite its impact on a high number of physiological and pathological conditions, many aspects of tissue-specific epigenetic regulation have so far remained unaddressed. Here, we aim to gather what is currently known about the effect of specific tissue microenvironments on NK cells and ILCs.

#### NK Cells and ILCs in Different Tissue Microenvironments

In an elegant transfer study Nussbaum et al. found, that the phenotype of adoptively transferred *Rorc*-fate map-positive (Rorc^fm+^) ILC3 was not determined by their tissue of origin but by the tissue they resided in after transfer: Rorc^fm+^ ILCs, which had homed to the spleen, acquired a NK-like phenotype with decent tumor-suppressive capacity, whereas Rorc^fm+^ ILCs which had migrated into the small intestine displayed an ILC3 phenotype and low anti-tumoral activity (100). This highlights, how distinct tissue microenvironments shape ILC diversity and functionality. This finding is underlined by the tissue-specific divergencies of group 1 ILC subpopulations with regards to the requirement of the lncRNA *Rroid*: *Rroid* regulates spleen, liver and lung NK cell and ILC1 homeostasis, however *Rroid* deficiency has no effect on the numbers of these cells within the salivary glands or the lamina propria of the small intestine, suggesting different requirements for the homeostasis of group 1 ILC in these organs (82). Along the same lines, the TF Hobit is crucial to the development of liver ILC1, whereas ILC1 in other organs are found in normal numbers in Hobit-deficient mice (87, 101). Another study, which investigated the transcriptomes of ILCs from different tissues, found that small intestine NK and small intestine ILC clustered closely together both transcriptionally and phenotypically, whereas clustering in other tissues was mainly driven by NK cell vs. ILC lineage commitment (102), again underlining the powerful effect of the small intestine microenvironment in shaping the local immune infiltrate. Little is known yet on which factors within these tissues are crucial for ILC plasticity and how they convey their signals. In an in-depth analysis of transcriptional and epigenetic properties of small intestine ILC, Gury-BenAri et al. demonstrated presumably microbiota-driven epigenetic alterations in small intestine ILC: in antibiotic-treated mice, siILC1 exhibited a modified epigenetic landscape marked by the loss of ILC1-specific enhancers like *Tcf7* or *Cd93* and the gain of ROR-bound, ILC3-associated enhancers (103). This suggests that microbiota-derived signals in the small intestine microenvironment might affect ILC transcriptional and epigenetic programs and thereby influence ILC plasticity. By contrast, homeostasis and survival of salivary gland ILC, which are endowed with a unique phenotype and transcriptome, is dependent on TGF-β abundance (104).

Besides the small intestine and salivary gland, the liver provides another unique immunological environment. Accordingly, singular NK cell and ILC subpopulations have been described both in mouse and human livers. For example, NK cells expressing the chemokine receptor CXCR6 are predominantly found within the liver and are present only in small percentages in peripheral blood (9, 11). It remains to be elucidated whether their organ-specific enrichment is caused solely by chemokine-induced liver homing or whether local differentiation takes place. Functionally, CXCR6^+^ NK cells have been shown to play a crucial role in hapten- and virus-induced memory and display functional and phenotypic differences to CXCR6^-^ NK cells (11, 105). Whereas the epigenetic properties of CXCR6^+^ liver-resident NK cells have not been sufficiently defined, another liver-resident, epigenetically primed NK cell subpopulation was recently described by Stary et al: CD49a+CD16- NK cells were predominantly found in the human liver as compared to peripheral blood and displayed distinct chromatin accessibility patterns associated with a higher susceptibility to cytokine stimuli and the potential to elucidate antigen-specific cytotoxicity against hepatitis A or B pulsed B cells (106). Further research will be required to shed light into the question, which components of the liver microenvironment (e.g. liver sinusoidal endothelial cells, hepatic stellate cells) lead to the development and/or enrichment of these specific subsets in the liver.

Another fascinating tissue microenvironment is offered by the uterus, where decidual NK cells (dNK cells) possess unique phenotypic and functional properties. Intriguingly, NK cell frequencies and functional properties undergo significant changes throughout the menstrual cycle and, even more pronouncedly, during pregnancy (107). Different stimuli, including demethylating agents, have been proposed to produce decidual-like NK cells, suggesting an epigenetic contribution to their development (108). Moreover, CpG island methylation patterns in uterine and decidual NK cells were found to be different from NK cells derived from breast or lymph nodes, but interestingly resembled breast cancer associated NK cells, indicating tissue-specific epigenetic remodeling (109). Intriguingly, Gamliel et al. observed a phenotypically and transcriptionally distinct NKG2C^high^ NK cell population in the decidua of multigravid women, which showed increased accessibility at the *Ifng* and *Vegfa* loci, accompanied by a higher propensity to IFN- γ and VEGF-A secretion upon cytokine stimulation, highlighting how temporary changes in the microenvironment, as they occur during pregnancy, can produce epigenetically primed novel NK cell subsets (110).

#### NK Cells and ILC in the Tumor Microenvironment

Unique microenvironments not only exist within healthy tissues, but also in pathological circumstances, most prominently within solid malignant tumors. It is well known that cancer tissues are often poorly infiltrated by NK cells and that tumor-infiltrating NK cells are functionally impaired (10). Since NK cell functionality is tightly regulated by epigenetic mechanisms, inhibitory signals transmitted by the tumor microenvironment (TME) are likely to also entail short-term or long-term epigenetic remodeling. A study investigating NK cell responses against breast cancer cells found that NK cells, which had been in contact with tumor cells for a while, switched from a tumoricidal state to a metastasis-promoting state (111). While the exact mechanism behind this functional switch remained unclear, it was associated with an upregulation of DNMTs within the NK cells, and the effect could be reversed by DNMT inhibition, suggesting an epigenetic contribution (111). Upon chronic TCR stimulation within the TME, CD8 T cells tend to exhibit features of exhaustion, marked by both phenotypic and functional alterations. Similarly, it was shown that chronic stimulation *via* the activating NKG2C receptor leads to an up-regulation of the exhaustion-associated molecules LAG-3 and PD-1 and to a decreased IFN- γ production in NK cells (112). Moreover, these chronically stimulated NK cells underwent significant epigenetic remodeling, with novel hypomethylated enhancers on genes associated with CD8 T cell exhaustion (112). This study was not carried out in a tumor context, but it is evident, that similar mechanisms *via* tumor-associated activating receptor ligands could lead to an epigenetic NK cell reprogramming within a TME rich in activating receptor ligands.

Apart from cell contact-dependent mechanisms, soluble ligands within the TME can exert inhibitory signals on invading immune cells. One of the most well described immunosuppressive cytokines often upregulated within the TME is transforming growth factor beta (TGF-β). The effects of TGF-β on tumor cells and immune cells are pleiotropic and especially the epigenetic consequences of TGF-β signaling are not yet fully understood. Donatelli et al. described a decreased expression of the activating receptor adaptor protein DAP12 in non-small cell lung cancer (NSCLC)- infiltrating NK cells, which was caused by the TGF-β-induced miRNA miR-183 (113). MiR183 targets DAP12 mRNA and decreases its levels, resulting in a decreased cytotoxic capacity of the affected NK cells (113). Of note, another miR-183 binding site was identified on the 3’ untranslated region (UTR) of the NKG2D ligands MICA and MICB, negatively regulating their expression and thereby NK cell recognition *via* NKG2D and possibly offering another mechanism of TGF-β mediated immune escape on the tumor cell side (114). Additionally, another TGF-β induced miRNA, miR-1245, has been shown to downregulate NKG2D levels on NK cells, again impeding target cell recognition (115). It can be assumed, that TGF-β-mediated signaling in NK cells relies on further modes of epigenetic remodeling, which have yet to be identified. Reports indicating a role for the HAT p300 in SMAD-mediated signaling downstream TGF-β highlight the potential role for histone modifications in this context (116, 117). Additionally, it has been described that NK cells undergo phenotypic and functional changes in TGF-β rich environments like the salivary gland, the obese liver, the uterus and certain tumors - a process often termed conversion into ILC-1 like cells due to the acquired phenotypic characteristics (like the upregulation of CD49a and downregulation of CD49b and Eomes) (104, 118, 119). This terminology however was challenged by a recent study employing single cell transcriptomic analysis and demonstrating a clear NK cell signature in CD49a^high^ cells derived from TGF-β signaling in the uterus and salivary gland (98). Independent of terminology, the phenotypic and functional conversion described here has been shown to inhibit NK cell anti-tumoral functionality (118). Conversely, TGF-β was recently demonstrated to be required for the maintenance of granzyme C expressing cytotoxic ILC1 in breast cancer tissues and lack of TGF-β signaling in these cells lead to an accelerated tumor growth (88). It will thus be intriguing to unravel the epigenetic components of the diverse effects downstream TGF-β in different cellular contexts. Further inhibitory factors in the TME like prostaglandin E2 (PGE2) or IDO have been described to severely affect NK cell functionality on multiple levels, but their short-term and long-term effects on the NK cell epigenetic landscape remain to be determined (120–123). A detailed understanding of how different TMEs affect NK cell epigenetic states in the short term and long term will be necessary, if we want to therapeutically enhance NK cell functionality within these complex inhibitory local environments.

## Concluding Remarks

Epigenetic mechanisms dictate crucial developmental steps and functional properties in NK cells. The epigenetic landscape, however, is not only influenced by cell-intrinsic signals, but it is also modified by cues from the microenvironment. The consequences of epigenetic remodeling can be long-lasting, as has been demonstrated in MCMV memory NK cells (59). However, not only activation and memory formation, but also exhaustion and dysfunction are epigenetically regulated: Philip et al. reported in 2017 that tumor-specific CD8 T lymphocytes acquire distinct dysfunction-related chromatin accessibility states throughout their time within the tumor, and in human CD8 T cells a chronic infection-induced epigenetic scarring was observed, which persisted long after the infection was resolved (124, 125). These findings demonstrate that external signals can lead to a permanent functional impairment in immune cells *via* epigenetic remodeling. NK cells have been found in a dysfunctional state within a number of solid tumors, which is one major impediment to the development of efficient NK cell-based therapies for non-hematologic cancers. Defining the microenvironmental cues, which shape NK cell functional states, and unraveling their epigenetic impact will thus be critical to the advancement of NK cell therapies. Despite the initial findings summarized in this review, many questions remain to be addressed. Novel techniques like CUT&Tag and CUTAC, which allow for analyses of histone modifications and chromatin accessibility in low cell numbers and with great resolution, are extending our possibilities to unravel epigenetic properties of small cell populations and in diverse tissues (126, 127). Moreover, single cell ATAC-seq will allow us to investigate the diversity of the accessible chromatin within different cell subpopulations (128). It will thus be exciting to investigate in depth how different microenvironments shape NK cell epigenetics – our understanding of these mechanisms will help us not only to better understand local inflammatory diseases but it will also allow us to better tailor NK cell-based therapies for different solid tumors.

## Author Contributions

The author confirms being the sole contributor of this work and has approved it for publication.

## Funding

GMW is funded by the Deutsche Forschungsgemeinschaft (DFG) Emmy Noether program (WI4927/2-1) and by the TUM Junior Fellows Fund (Technical University of Munich).

## Conflict of Interest

The author declares that the research was conducted in the absence of any commercial or financial relationships that could be construed as a potential conflict of interest.

## Publisher’s Note

All claims expressed in this article are solely those of the authors and do not necessarily represent those of their affiliated organizations, or those of the publisher, the editors and the reviewers. Any product that may be evaluated in this article, or claim that may be made by its manufacturer, is not guaranteed or endorsed by the publisher.
